# Effects of Telerehabilitation Based on Motion Recognition Technology on Exercise Endurance of Patients With Non–Small Cell Lung Cancer After Surgery: Single-Center, Prospective, Open-Label, Randomized Controlled Trial

**DOI:** 10.2196/82447

**Published:** 2026-06-29

**Authors:** Ling Chen, Xiaoyi Xu, Xiaoliang Yang, Siying Long, Na Chen, Zifan Li, Tingting Yang, Qianru Li, Meng Li, Hai Zhong, Guozhi Huang, Qing Zeng

**Affiliations:** 1 Center of Rehabilitation Medicine, Zhujiang Hospital, Southern Medical University Guangzhou, Guangdong Province China; 2 School of Rehabilitation Sciences, Southern Medical University Guangzhou, Guangdong Province China; 3 GuangDong Engineering Technology Research Center of Brain Function Assessment and Neuroregulation Rehabilitation Guangzhou, Guangdong Province China; 4 Institute of Exercise and Rehabilitation Science, Zhujiang Hospital, Southern Medical University Guangzhou, Guangdong Province China

**Keywords:** non–small cell lung cancer, artificial intelligence, motion recognition, telerehabilitation, exercise endurance

## Abstract

**Background:**

Non–small cell lung cancer (NSCLC) accounts for approximately 85% of primary pulmonary neoplasms. Complete surgical removal remains the cornerstone of curative therapy, yet it frequently diminishes residual lung function and exercise tolerance. Structured, center-based rehabilitation hastens physiological recovery, but conventional schemes rarely deliver continuous, patient-specific monitoring. Remote, digitally delivered exercise overcomes logistical obstacles; however, the lack of real-time quality assurance curtails effectiveness. Wearable motion capture platforms that provide millimeter-precise kinematic data and instantaneous biomechanical feedback can close this supervisory void by confirming movement accuracy and issuing immediate corrective prompts. Whether this technologically augmented telerehabilitation yields clinically relevant improvements in postoperative exercise capacity after NSCLC resection remains inadequately established.

**Objective:**

This study aims to find out whether motion capture–enabled telerehabilitation can enhance exercise endurance and functional recovery following NSCLC resection, thereby filling a critical void in postoperative care pathways.

**Methods:**

We performed a single-center, parallel-arm randomized trial in individuals who had completed curative lung resection for NSCLC and satisfied every enrollment criterion. Following randomization, eligible patients were assigned either to a technology-enhanced telerehabilitation protocol incorporating live motion sensing or to a WeChat-based regimen without that feedback layer. Each program spanned 4 weeks. Assessments—comprising exercise endurance, spirometric variables, health-related quality of life, and daily physical activity—were collected at discharge and repeated 4 weeks after intervention.

**Results:**

No exercise-related adverse events occurred during the 4-week intervention. Compared with conventional video-guided training, the motion recognition group demonstrated significantly greater improvements in exercise tolerance, pulmonary function, selected functional mobility outcomes, and quality of life. The intervention group achieved a greater increase in 6-minute walk distance (mean difference 32 meters, 95% CI 5.40-58.60; *P*=.02). Significant between-group differences were also observed in forced vital capacity (mean difference 476.4 mL; *P*=.02), forced expiratory volume in 1 second (mean difference 346.0 mL; *P*=.04), FEV_1_/FVC ratio (mean difference 12.8%; *P*=.03), and peak expiratory flow (mean difference 70.8 L/minute; *P*=.002). For functional mobility, the intervention group showed superior improvement in the Timed Up and Go test (mean difference −1.63 seconds; *P*=.04), whereas no significant between-group differences were found in other mobility measures. Quality of life outcomes favored the motion recognition group, with greater improvements in physical well-being, functional well-being, additional concerns–lung subscale, and total Functional Assessment of Cancer Therapy–Lung score (mean difference 13.00; *P*<.001). Program adherence was higher in the intervention group (72% vs 40%; *P*=.02).

**Conclusions:**

Four-week motion capture–guided telerehabilitation yielded clinically meaningful gains in aerobic endurance, spirometric indices, ambulatory capacity, and global health–related quality of life for patients recovering from NSCLC surgery, underscoring its usefulness as a safe and effective remote care strategy.

**Trial Registration:**

Chinese Clinical Trial Registry ChiCTR2500113139; https://www.chictr.org.cn/showprojEN.html?proj=270991

## Introduction

Lung cancer continues to rank among the leading causes of malignant disease worldwide, exerting a disproportionate share of global oncological morbidity [1]. Global cancer statistics for 2022 recorded roughly 2.48 million incident cases—about 1 in 8 newly diagnosed tumors [2]. Non–small cell lung cancer (NSCLC) constitutes approximately 85% of this total [3]. Curative-intent surgical resection remains the primary therapeutic modality; yet, a substantial proportion of recipients subsequently experience pronounced reductions in aerobic capacity and ventilatory efficiency, translating into markedly lower health-related quality of life [4]. Exercise-based rehabilitation has repeatedly been demonstrated to augment cardiorespiratory fitness, elevate peak oxygen uptake, and enhance both muscular strength and endurance [5]. Physical training is therefore regarded as a cornerstone of postoperative recovery after NSCLC resection. Randomized evidence indicates that regimented exercise improves exertional tolerance, fortifies respiratory musculature [6], and confers psychological benefit [7,8]. Traditional, center-based programs—usually conducted in hospitals or specialized rehabilitation units—continue to confront entrenched barriers. Uneven geographic distribution of trained therapists leaves numerous primary care settings without access to specialist services, impeding timely intervention. Logistical burdens—lengthy travel, inflexible scheduling, and direct and indirect costs—further erode participation, while repeated clinic visits impose additional physical and psychosocial strain on patients and caregivers.

Digital telerehabilitation—encompassing videoconferencing, smartphone apps, and immersive virtual reality—overcomes spatial and temporal barriers to specialist care. Recent studies have suggested that eHealth-supported pulmonary rehabilitation may improve musculoskeletal symptoms and quality of life in patients with post–COVID-19 condition [9]. Furthermore, structured telemedicine-based pulmonary rehabilitation protocols have been developed for individuals with post–COVID-19 condition and persistent respiratory symptoms, reflecting ongoing efforts to establish remote rehabilitation approaches.[10]. In addition, recent systematic reviews and meta-analyses have confirmed that pulmonary rehabilitation significantly improves exercise capacity, pulmonary function, and quality of life in individuals with long COVID-19 syndrome, further reinforcing the clinical value of structured respiratory rehabilitation programs [11]. Evidence also links these remote interventions to prolonged survival, improved quality of life, and heightened self-efficacy in lung-cancer survivors [12]. Park et al [12] deployed the Smart Aftercare App to deliver postoperative exercise guidance and reported significantly fewer outpatient encounters among active users than among nonusers. Despite such benefits, conventional telerehabilitation platforms typically depend on asynchronous demonstration clips or synchronous verbal coaching, neither of which can supply real-time biomechanical feedback or quantitative motion assessment. This deficit jeopardizes movement fidelity and complicates the standardization of exercise execution.

Action recognition platforms integrate computer vision, wearable sensors, and machine learning classifiers to deliver millimeter-level appraisal of human kinematics. Over the past decade, this convergence has fueled growing interest in digitally mediated rehabilitation. He et al [13] reported that exercise regimens augmented by movement capture analytics yield gains in lean mass, functional capacity, and health-related quality of life that rival both center-based therapy and conventional tele-exercise. Chae and coworkers [14] created an artificial intelligence (AI)–driven fitness application that issues instantaneous postural cues during exercise. The participants who received algorithmic feedback improved their action accuracy by more than 32% and demonstrated higher execution consistency than those who followed the standard video instructions [14]. Extending this paradigm, our group previously conducted a randomized controlled trial in adults with chronic nonspecific low back pain. After allocation to either an AI-guided, real-time feedback group or a traditional video-only group, the AI cohort exhibited a pronounced reduction in pain intensity and a concomitant hypertrophic response in the deep core musculature within 4 weeks [15].

Based on this, this study aims to explore the application value of motion recognition technology in remote rehabilitation after NSCLC lung cancer surgery. By providing objective and quantifiable motion data, it enables precise evaluation of patients’ rehabilitation progress, thereby filling the gap in existing research, promoting innovative development of postoperative rehabilitation models for lung cancer, and delivering higher-quality rehabilitation services to patients.

## Methods

### Study Design

This was a single-center, prospective, open-label, randomized controlled trial. We used an offline approach to screen adult patients who underwent radical lung cancer surgery in the Department of Thoracic Surgery at Zhujiang Hospital, Southern Medical University, between June 2024 and January 2025, and the study protocol adhered to the CONSORT-EHEALTH (V 1.6; Consolidated Standards of Reporting Trials of Electronic and Mobile Health Applications and Online Telehealth) checklist (Multimedia Appendix 1). The study was prospectively registered in the Chinese Clinical Trial Registry (ChiCTR2500113139).

### Sample Size Calculation

The sample size was determined based on the primary outcome—6-minute walk distance (6MWD). Previous studies have demonstrated that telerehabilitation can significantly improve 6MWD in patients following lung cancer surgery, and the minimal clinically important difference for 6MWD in this population has been reported to be 50 meters [16]. Accordingly, we hypothesized that the motion capture telerehabilitation intervention would yield a clinically meaningful improvement of 50 meters in 6MWD compared with the control group. Assuming a 2-sided α value of .05 and a power of 80%, the required sample size was calculated to be 23 participants per group. To account for an anticipated dropout rate of 20%, the sample size was inflated to 29 participants per group. Therefore, a total of 58 participants were required for this study.

### Ethical Considerations

The clinical study was conducted in accordance with the Declaration of Helsinki and China regulations on clinical research and has been approved by the medical ethics committee. All participants signed informed consent forms. Prior to the formal initiation of this study, it was approved by the Ethics Committee of Zhujiang Hospital, Southern Medical University (approval 2024-KY-327-01, Medical Research Registration and Filing Information System Registration MR-44-24-052709). The study has been registered in the Chinese Clinical Trial Registry (ChiCTR2500113139). The study was conducted by trained investigators, who were responsible for patient recruitment, data collection, and test administration. The data collected included patients’ basic information, clinical data, exercise tolerance, pulmonary function, quality of life scales, mobility, treatment adherence, and adverse event incidence. For patients capable of completing the paper-based questionnaire independently, the investigators provided standardized questionnaires and guided them through the completion process. For patients unable to complete the questionnaire independently due to physical conditions or other reasons, the investigators conducted oral inquiries and recorded responses accordingly. During the recording process, the investigators verified information with patients to ensure data accuracy and completeness, and completed the questionnaire after confirming no errors. Throughout the research process, the investigators strictly adhered to ethical guidelines, safeguarding patient privacy and data security. All collected data were processed anonymously and used solely for research purposes, with no disclosure to third parties without patient consent. Participants received complimentary health education resources and were granted access to the AI-driven telerehabilitation platform throughout the intervention phase, while no monetary incentives were offered to participants for taking part in the study.

### Participant Selection and Recruitment

The study selected patients with NSCLC who underwent thoracic surgery and were hospitalized at Zhujiang Hospital of Southern Medical University from June 2024 to January 2025 as the research participants. A total of 58 eligible patients were enrolled. The research team provided comprehensive and detailed explanations to all enrolled patients and their families, covering the purpose, specific content, and significance of the study.

### Eligibility Standards

Inclusion criteria included (1) histopathologic diagnosis of NSCLC per National Comprehensive Cancer Network Guidelines 2024.V4 [17], (2) age 18-75 years, (3) cognitive competence adequate for assessment and exercise adherence, and (4) completed radical resection (lobectomy, segmentectomy, or wedge) with stable postoperative vital signs. Exclusion criteria included (1) clinically significant impairment of cardiac, hepatic, or renal function precluding exercise; (2) uncontrolled postoperative complications such as pneumonia, atelectasis, pleural effusion, or respiratory failure; (3) concurrent malignancy likely to influence recovery; (4) enrollment in another interventional study within the preceding 90 days; (5) enrollment in another interventional study within the preceding 90 days; and (6) inability to use a smartphone or absence of reliable internet connectivity required for motion capture telerehabilitation.

### Randomization and Blinding

Randomization was conducted using a computer-generated random number sequence in a 1:1 ratio. Simple randomization without blocking or stratification was applied. The allocation sequence was generated by an independent researcher (QZ) and concealed using sequentially numbered, opaque, and sealed envelopes to ensure allocation concealment. All researchers involved in patient recruitment or data collection will not be involved in the generation of the random sequence and the preparation and production of the random allocation concealment envelopes. The primary and all secondary outcome measures were collected offline through face-to-face interviews.

### Interventions

#### Telerehabilitation App Incorporating Action Recognition Technology

**Figure 1:**
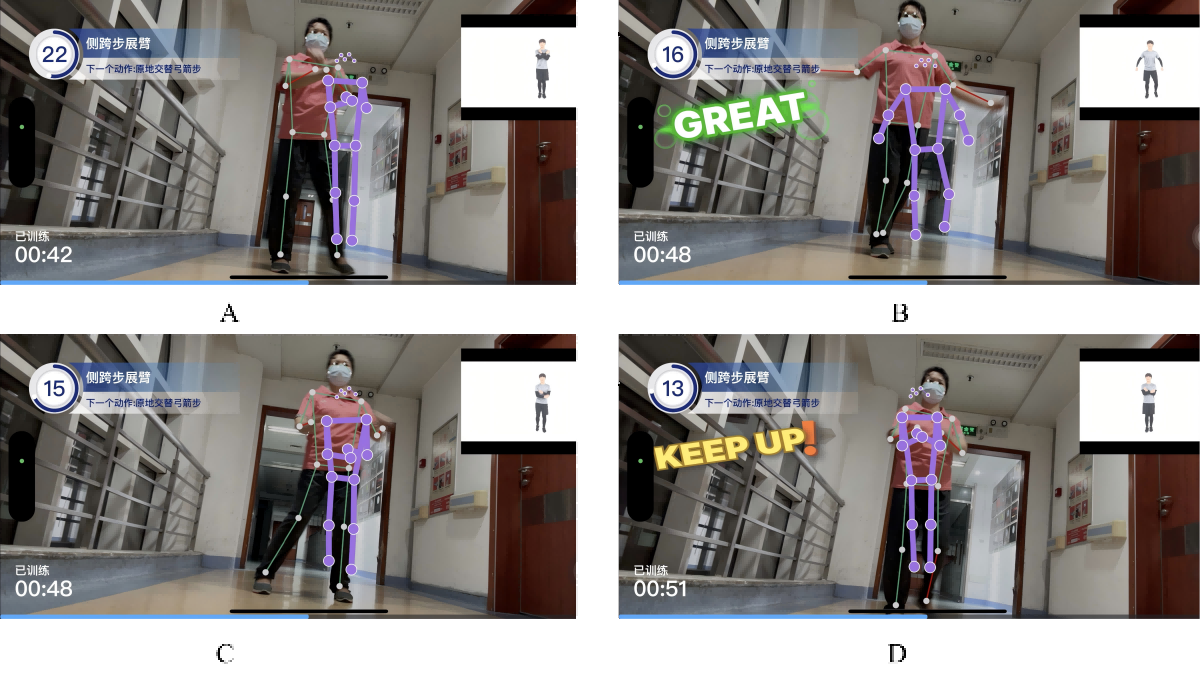
Training diagram of experimental group. (A) The system demonstrates the “Running in Place” exercise, displaying real-time skeletal motion tracking, exercise guidance, and countdown timing. (B) The system demonstrates the “Side Steps with Arm Extension” exercise with posture recognition and movement feedback during training. (C) The system demonstrates the “Alternating Lunges in Place” exercise while continuously tracking joint trajectories and exercise performance. (D) The system demonstrates the “Chest Expansions With Heel Touches” exercise with synchronized skeletal overlay and live exercise instructions.

The intervention group trained via the Rehabilitation Exercise Prescription app, whose core engine is a skeleton-driven motion recognition module. The software perpetually maps 3D coordinates of anatomical landmarks and then superimposes a chromatic skeletal overlay on the live video feed to log every trajectory. As depicted in Figure 1A-D, the user interface foregrounds the active exercise label together with a decrementing timer; preparatory cues smooth transitions between successive movements.

Instant textual feedback—both corrective and motivational—is pushed immediately after each repetition to sustain engagement. High fidelity is achieved through deep learning classifiers that pit the participant’s kinematic signature against a gold-standard template, flagging deviations and confirming when performance satisfies therapeutic thresholds.

#### Remote Rehabilitation Without Action Recognition

**Figure 2:**
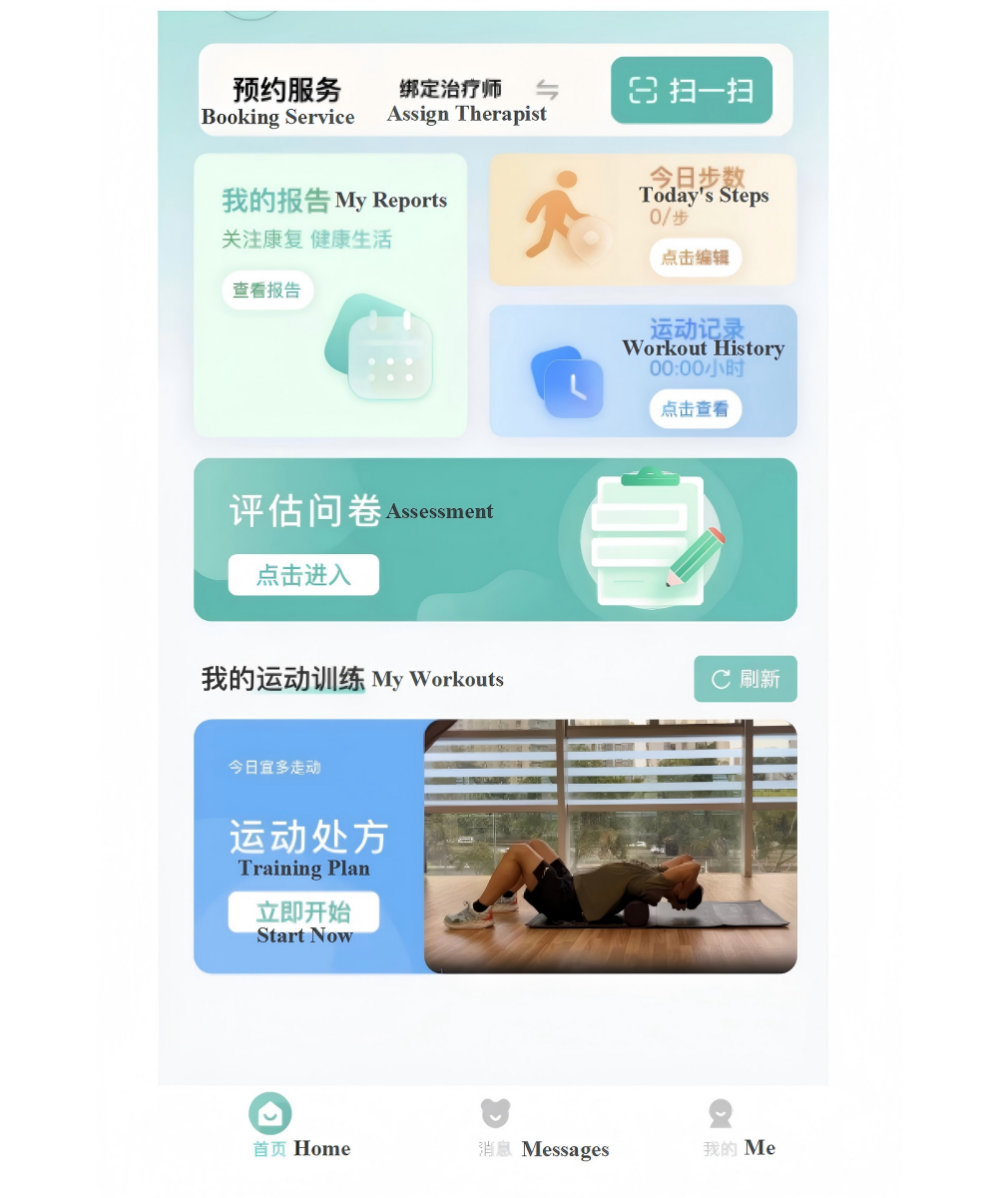
Home page of WeChat mini program.

Members of the control group followed a home exercise protocol hosted on a WeChat mini program (Figure 2). The module provided only asynchronous video demonstrations; it lacked skeleton-tracking analytics, instantaneous correction, and any mechanism to log or verify completion.

#### Assessment

All assessments and questionnaire surveys were conducted in person through face-to-face evaluation sessions. The assessment included the following:

1. *General data of patients*: sociodemographic variables: full name, age, sex, stature, body mass, BMI, education level, occupation, tobacco exposure, habitual physical activity patterns, medical insurance category, and habitual activity levels.
2. *Disease-specific metrics*: overall length of stay, postoperative inpatient days, adjuvant chemotherapy or radiotherapy use, histologic subtype, and tumor-node-metastasis stage according to the IASLC eighth edition guidelines and surgical approach.
3. *Exercise endurance*: 6-minute walk test (6MWT, Borg scale).
4. *Pulmonary function*: forced vital capacity (FVC), forced expiratory volume in 1 second (FEV_1_), the FEV_1_/FVC ratio, and peak expiratory flow (PEF).
5. *Quality of life indicators*: Functional Assessment of Cancer Therapy–Lung v4.0 (FACT-L) [18]: the core covers four dimensions [19]: physical well-being (PWB, 7 items)—energy, pain, and fatigue [20]; social/family well-being (SWB, 7 items)—relationships and perceived support [18]; emotional well-being (EWB, 6 items)—anxiety and depressive symptoms; functional well-being (FWB, 7 items)—daily activities and mobility [21].
6. *Measurement of functional mobility*: Five-times Sit-to-Stand Test (FTSST) [22], 30-second Chair-Stand Test (30-second CST) [23], Timed Up and Go (TUG) [24], Single-leg stance [25], and 2-minute step test [26].
7. *Exercise adherence*: individuals completing ≥3 sessions per week were deemed adherent; those completing <3 sessions per week were considered nonadherent.

The pulmonary function test graph and the 6MWT graph are shown in Multimedia Appendix 2.

### Exercise Program Development and Implementation

Table 1 presents the structured components of the postoperative home rehabilitation program implemented in the experimental group. The intervention was organized according to specific time points, including discharge day, 4 weeks after discharge, and the follow-up assessment. It outlines the intervention items, detailed content, implementation methods, and prescribed frequency and duration.

**Table 1:** Contents of postoperative home rehabilitation of patients in the experimental group.

Intervention timing and item		Specific content	Implementation method		Frequency/duration	
On the day of discharge						
	Discharge assessment	Assess exercise tolerance, pulmonary function, daily activity ability, and quality of life	In-hospital assessment		Single session, approximately 60 minutes	
From discharge to 4 weeks				Rehabilitation Exercise Prescription app (experimental)/WeChat mini-program (control)		3-5 times per week, 20-30 minutes per session
	Aerobic training	Stationary jogging Jumping jacks High-knee runs				
	Strength training	Side-leg raises Body-weight squats Step-ups Prone planks				
	Flexibility training	Seated forward folds Static bow stances				
From the day of discharge to 4 weeks after discharge						
	Follow-up management	A dedicated postdischarge support unit—consisting of thoracic surgeons, rehabilitation physicians, nurses, and therapists—monitored each participant through WeChat. The group sent session reminders, delivered immediate feedback, and addressed emerging questions. Outcome evaluations were scheduled 4 weeks (2 days) after discharge to gauge intervention efficacy.	WeChat + phone calls		2-3 times a week	
4 weeks (2 days) after discharge						
	Outcome assessment	Assess all outcomes	Outpatient assessment		Single session, approximately 60 minutes	

On the day of discharge, participants underwent an in-hospital discharge assessment to evaluate exercise tolerance, pulmonary function, activities of daily living, and quality of life. During the 4-week exercise after discharge, the intervention consisted of structured exercise training delivered through a digital platform, incorporating aerobic, strength, and flexibility exercises.

### Follow-Up

Remote supervision and follow-up management were conducted by a multidisciplinary team via online communication tools such as the WeChat app and telephone contact to enhance adherence and provide timely feedback. At 4 weeks after discharge, outcome assessments were performed in the outpatient setting using the same evaluation domains as baseline.

### Statistical Analyses

All analyses were performed in SPSS (version 26.0; IBM Corp). Normally distributed continuous data are presented as mean (SD); group differences and pre-to-post changes were examined with independent-samples and paired *t* tests, respectively. Non–normally distributed variables are summarized by medians; between-group contrasts were evaluated using the Mann-Whitney *U* test, and within-group shifts via Wilcoxon signed-rank tests. Categorical variables are described with counts and percentages, and baseline comparability was verified by chi-square analysis. The last available primary end point value was carried forward for withdrawals. Missing data mechanisms guided handling: missing completely at random observations were listwise-deleted, missing at random values were multiply imputed, and missing not at random scenarios were explored with sensitivity analyses using pattern mixture or selection models.

## Results

### Enrollment

**Figure 3:**
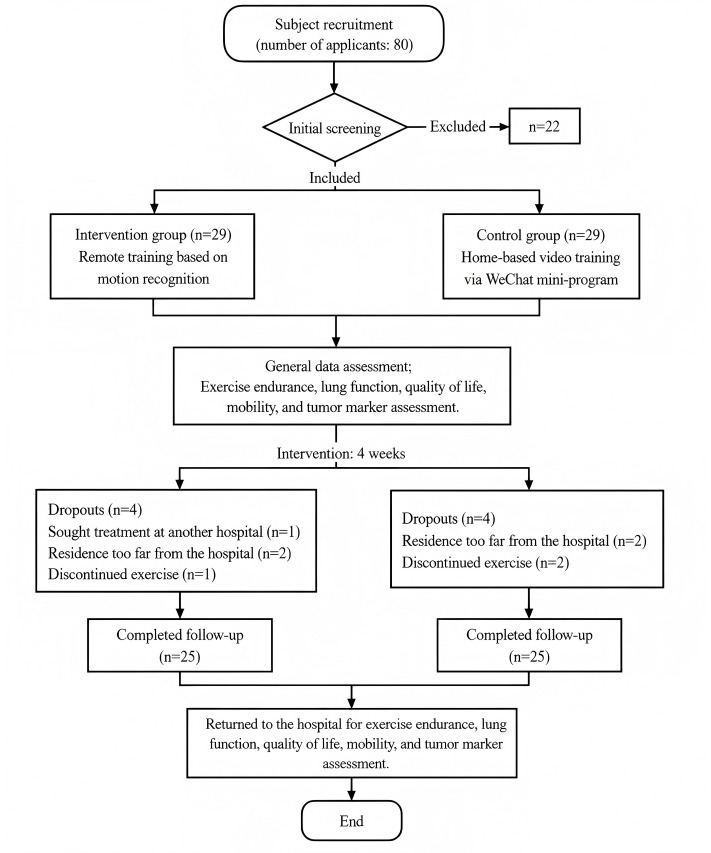
Flowchart of study process.

A total of 80 applicants were initially recruited and underwent preliminary screening. Following the eligibility assessment, 22 individuals were excluded, and the remaining participants were included in the trial. Fifty-eight patients were randomized (29 per group). During follow-up, 4 participants in each group withdrew from the study. In the intervention group, reasons for dropout included seeking treatment at another hospital, residing too far from the hospital, and discontinuation of exercise. In the control group, dropouts were due to relocation far from the hospital or discontinuation of exercise. Ultimately, 25 participants in each group completed the follow-up assessment. Consequently, 50 participants (25 experimental and 25 control) provided complete data for the final analysis (Figure 3).

### General Data of Patients

A total of 50 patients with lung cancer were enrolled and randomly allocated to the experimental group (n=25) and the control group (n=25). The mean age was 54.56 (SD 12.12) years in the experimental group and 56.96 (SD 11.02) years in the control group, with no statistically significant difference between groups (*P*=.62). In terms of sex distribution, 40% (10/25) of the participants in the experimental group and 48% (12/25) in the control group were male, and the difference was not statistically significant (*P*=.39). No significant differences were observed between the 2 groups with respect to height, body weight, or BMI (all *P*>.05). Similarly, baseline characteristics including educational attainment, occupational status, and exercise habits were evenly distributed between groups, with no significant between-group differences (all *P*>.05), suggesting comparability at baseline (Table 2).

**Table 2:** Comparison of demographic characteristics between the 2 groups.

Indicators			Experimental group (n=25)		Control group (n=25)		*t* test (*df*)		*P* value
Sex, n (%)							0.776 (1)		.39
	Male	10 (40)		12 (48)					
	Female	15 (60)		13 (52)					
Age (years), mean (SD)			54.56 (12.12)		56.96 (11.02)		–0.495 (48)		.62
Height (cm), mean (SD)			162.24 (7.03)		162.52 (5.83)		–0.153 (48)		.80
Body weight (kg), mean (SD)			60.04 (11.31)		58.5 (6.62)		–0.583 (48)		.56
BMI (kg/m^2^), mean (SD)			22.77 (3.68)		22.19 (2.59)		0.646 (48)		.52
Degree of education, n (%)							5.219 (3)		.16
	Primary school and below	9 (36)		5 (20)					
	Junior high school	4 (16)		8 (32)					
	Technical secondary school/high school	9 (36)		5 (20)					
	Bachelor degree or above	3 (12)		7 (28)					
Occupations, n (%)							1.666 (6)		.95
	Student	1 (4)		0					
	Workers	4 (16)		4 (16)					
	Farmer	2 (8)		3 (12)					
	Staff member	2 (8)		2 (8)					
	Self-employed persons	2 (8)		3 (12)					
	Retirees	10 (40)		10 (40)					
	Unemployment	4 (12)		3 (12)					
Habit of exercise, n (%)							0.125 (1)		.72
	Have	6 (24)		4 (16)					
	No	19 (76)		21 (84)					

There were no significant differences between the 2 groups in smoking history, medical insurance type, history of chemoradiotherapy, pathological type, surgical method, length of hospital stay distribution, or postoperative hospital stay duration (all *P≥*.05). The majority of participants in both groups had stage I lung cancer, and all patients were pathologically confirmed at stage I, indicating homogeneity in disease severity. Adenocarcinoma was the predominant pathological type, accounting for 92% in the experimental group and 100% in the control group. Regarding surgical procedures, most patients underwent wedge resection or segmentectomy, with comparable distributions between groups. Postoperative hospital stay was similar between the 2 groups, with a mean duration of 6.08 (SD 0.60) days in the experimental group and 4.84 (SD 0.48) days in the control group (*P*=.10). Overall, baseline clinical and perioperative characteristics were balanced between groups (Table 3).

**Table 3:** Comparison of disease-related data between the 2 groups.

Indicators		Experimental group (n=25)	Control group (n=25)	Test value		*P* value	
History of smoking, n (%)					0.087 (1)^a^		.76
	Have	8 (32)	10 (40)				
	No	17 (68)	15 (60)				
Types of medical insurance, n (%)					1.167 (3)^a^		.76
	Out of pocket	3 (12)	1 (4)				
	Local medical insurance	10 (48)	11 (40)				
	Provincial nonlocal medical insurance	10 (40)	13 (52)				
	Cross-provincial medical insurance	1 (4)	1 (4)				
History of chemoradiotherapy, n (%)					0.000 (2)^a^		>.99
	Radiation therapy	0 (0)	0 (0)				
	Chemotherapy	1 (4)	2 (8)				
	No	24 (96)	23 (92)				
Types of pathology, n (%)					0.521 (1)^a^		.47
	Adenocarcinoma	23 (92)	25 (100)				
	Squamous cell carcinoma	2 (8)	0 (0)				
Pathological staging, n (%)					—^b^		—
	Phase 1	25 (100)	25 (100)				
	Phase 2	0 (0)	0 (0)				
	Phase 3	0 (0)	0 (0)				
Surgical methods, n (%)					0.466 (2)^a^		.50
	Lobectomy of lung	7 (28)	4 (16)				
	Wedge resection/segmentectomy	18 (72)	21 (84)				
The average length of stay (days), n (%)					–1.556 (48)^c^		.12
	≤5	3 (12)	5 (20)				
	5-10	13 (52)	15 (60)				
	≥10	9 (36)	5 (20)				
Postoperative hospital stay, mean (SD)		6.08 (0.60)	4.84 (0.48)	–1.650 (48)^c^		.10	

^a^Chi-square test.

^b^Not applicable.

^c^*t* test.

### Comparison of Outcome Index Results

#### Related Indexes of Exercise Endurance Compared Between 2 Groups

At baseline, 6MWD and Borg fatigue scores were comparable across groups (*P*>.05). After 4 weeks, both groups showed significant within-group improvements in the 6MWD. The experimental group increased from a mean of 399.00 (SD 67.92) to 507.00 (SD 62.23) meters (*T*=–7.40; *P*<.001). The control group also improved from 415.12 (SD 73.13) to 460.96 (SD 71.24) meters (*T*=–3.39; *P*<.001). The between-group comparison at 4 weeks revealed a statistically significant difference favoring the motion capture cohort (mean difference 32 meters, 95% CI 5.40- 58.60; *T*=2.43; *P*=.02). For perceived exertion, both groups demonstrated significant reductions in Borg scores following the intervention. The experimental group decreased from 2.12 (SD 0.93) to 0.96 (SD 0.87; *T*=3.63; *P*<.001), while the control group decreased from 2.32 (SD 0.80) to 1.36 (SD 0.94; *T*=3.67; *P*<.001). However, the between-group difference for the change in Borg-perceived exertion scores was not statistically significant (mean difference –0.40, 95% CI –0.88 to 0.08; *T*=–1.65; *P*=.10). Complete data are provided in Table 4.

**Table 4:** Comparison of exercise endurance indexes between the 2 groups before and after intervention.

Index group			Pre-exercise, mean (SD)		Postexercise, mean (SD)		Between-group difference (95% CI)			*t* test (*df*)			*P* value^a^			Cohen *d*	
6MWD^b^								32.00 (5.40-58.60)									0.69
	Experimental group	399.00 (67.92)		507.00 (62.23)					–7.40 (24)			<.001					
	Control group	415.12 (73.13)		460.96 (71.24)					–3.39 (24)			<.001					
	*t* test (*df*)	–0.81 (48)		2.43 (48)					—^c^			—					
	*P* value	.42		.02					—			—					
Borg score								–0.40 (–0.88 to 0.08)									0.45
	Experimental group	2.12 (0.93)		0.96 (0.87)					3.63 (24)			<.001					
	Control group	2.32 (0.80)		1.36 (0.94)					3.67 (24)			<.001					
	*t* test (*df*)	–0.87 (48)		–1.65 (48)					—			—					
	*P* value	.38		.10					—			—					

^a^The significance level was set at *P*<.05.

^b^6MWD: 6-minute walk distance.

^c^Not applicable.

#### Pulmonary Function Indexes of the 2 Groups Compared

At baseline, pulmonary function measures were comparable between groups (*P*>.05). After 4 weeks, every spirometric variable improved more in the motion capture cohort than in controls (all *P*<.05). The control group did not show significant within-group changes in any of these measures (all *P*≥.05). For FVC, the experimental group increased from a mean of 1716.40 (SD 639.30) to 2576.80 (SD 629.54) mL (*T*=–7.14; *P*<.001), while the control group showed no significant change (2106.40, SD 823.67 mL to 2100.40, SD 742.77 mL; *T*=0.96; *P*=.35). The between-group comparison revealed a statistically significant difference favoring the motion capture cohort (mean difference 476.4 mL, 95% CI 85.2-867.6; *T*=2.45; *P*=.02). For FEV_1_, the experimental group improved from 1294.80 (SD 413.62) to 2029.20 (SD 526.79) mL (*T*=–8.74; *P*<.001), compared with minimal change in the control group (1462.28, SD 629.17 mL to 1683.20, SD 647.47 mL; *T*=–1.83; *P*=.08). The between-group difference was statistically significant (mean difference 346.0 mL, 95% CI 9.8-682.2; *T*=2.07; *P*=.04). For the FEV_1_/FVC ratio, the experimental group increased from 69.88% (SD 20.46) to 91.60% (SD 22.14; *T*=–3.55; *P*<.001), while the control group showed no significant improvement (72.04%, SD 18.30 to 78.80%, SD 18.01; *T*=–1.31; *P*=.20). The between-group comparison favored the experimental group (mean difference 12.8%, 95% CI 1.3-24.3; *T*=–2.15; *P*=.03). For PEF, the experimental group improved from 130.58 (SD 73.59) to 230.21 (SD 81.66) L/minute (*T*=4.23; *P*<.001), whereas the control group showed no significant change (170.04, SD 70.16 to 159.36, SD 62.26 L/minute [*T*=0.48; *P*=.63]). The between-group difference was statistically significant (mean difference 70.8 L/minute, 95% CI 26.4-115.2; *T*=–3.15; *P*=.002). Complete data are presented in Table 5.

**Table 5:** Comparison of pulmonary function indexes between the 2 groups before and after intervention.

Index and group		Pre-exercise, mean (SD)	Postexercise, mean (SD)	Between-group difference (95% CI)		*t* test (*df*)		*P* value^a^		Cohen *d*	
FVC^b^					476.00 (85.20-867.60)						0.71
	Experimental group	1716.40 (639.30)	2576.80 (629.54)			–7.14 (24)		<.001			
	Control group	2106.40 (823.67)	2100.40 (742.77)			0.96 (24)		.35			
	*t* test (*df*)	–1.87 (48)	2.45 (48)			—^c^		—			
	*P* value	.07	.02			—		—			
FEV_1_^d^					346.0 (9.80-682.2)						0.62
	Experimental group	1294.80 (413.62)	2029.20 (526.79)			–8.74 (24)		<.001			
	Control group	1462.28 (629.17)	1683.20 (647.47)			–1.83 (24)		.08			
	*t* test (*df*)	–0.73 (48)	2.07 (48)			—		—			
	*P* value	.47	.04			—		—			
FEV_1_/FVC^e^					12.80 (1.3-24.3)						0.58
	Experimental group	69.88 (20.46)	91.60 (22.14)			–3.55 (24)		<.001			
	Control group	72.04 (18.30)	78.80 (18.01)			–1.31 (24)		.20			
	*t* test (*df*)	–0.28 (48)	–2.15 (48)			—		—			
	*P* value	.78	.03			—		—			
PEF^f^					70.8 (26.4-115.2)						0.95
	Experimental group	130.58 (73.59)	230.21 (81.66)			4.23 (24)		<.001			
	Control group	170.04 (70.16)	159.36 (62.26)			0.48 (24)		.63			
	*t* test (*df*)	–1.89 (48)	–3.15 (48)			—		—			
	*P* value	.06	.002			—		—			

^a^The significance level was set at *P*<.05.

^b^FVC: forced vital capacity.

^c^Not available.

^d^FEV_1_: forced expiratory volume in 1 second.

^e^FEV_1_/FVC: The ratio between FEV_1_ and FVC.

^f^PEF: peak expiratory flow.

#### Activity Ability Indexes of the 2 Groups Compared

At baseline, activity-capacity indices were comparable across groups (all *P*>.05). After 4 weeks, both groups demonstrated significant within-group improvements in most functional mobility measures, with the motion capture group showing superior gains in the TUG test. For the TUG test, the experimental group improved from a mean of 13.11 (SD 4.39) to 9.95 (SD 2.41) seconds (*T*=3.46; *P*<.001), while the control group improved from 12.78 (SD 3.35) to 11.58 (SD 3.04) seconds (*T*=2.42; *P*=.02). The between-group comparison revealed a statistically significant difference favoring the motion capture cohort (mean difference –1.63 seconds, 95% CI –3.19 to –0.07; *T* =–2.11; *P*=.04). For Stand on 1 foot, the experimental group improved from 24.79 (SD 19.71) to 43.72 (SD 31.59) seconds (*T*=4.14; *P*<.001), and the control group improved from 38.36 (SD 79.30) to 49.68 (SD 58.19) seconds (*T*=2.52; *P*=.01). However, the between-group difference was not statistically significant (mean difference –5.96 seconds, 95% CI –33.2 to 21.3; *T*=–0.44; *P*=.66). For the 30-second CST, the experimental group improved from 9.52 (SD 3.55) to 13.96 (SD 4.94) repetitions (*T*=–5.73; *P*<.001), and the control group improved from 11.16 (SD 3.88) to 12.68 (SD 3.00) repetitions (*T*=–2.63; *P*=.02). The between-group difference did not reach statistical significance (mean difference 1.28 repetitions, 95% CI –1.18 to 3.74; *T*=0.78; *P*=.43). For the FTSST, the experimental group improved from 13.56 (SD 6.78) to 9.32 (SD 2.90) seconds (*T*=3.79; *P*<.001), and the control group improved from 11.03 (SD 3.00) to 9.48 (SD 2.24) seconds (*T*=2.35; *P*=.02). The between-group difference was not statistically significant (mean difference–0.16 seconds, 95% CI –1.67 to 1.35; *T*=–0.25; *P*=.80). For the 2-minute step test, the experimental group improved from 1832.56 (SD 41.46) to 179.84 (SD 38.27) steps (*T*=–6.10; *P*<.001), and the control group improved from 143.88 (SD 33.74) to 177.48 (SD 30.25) steps (*T*=–4.82; *P*<.001). The between-group difference was not statistically significant (mean difference 2.36 steps, 95% CI –17.8 to 22.5; *T*=0.24; *P*=.81). Complete data are provided in Table 6.

**Table 6:** Comparison of indicators of activity ability between the 2 groups before and after intervention.

Index and group		Pre-exercise, mean (SD)	Postexercise, mean (SD)	Between-group difference (95% CI)		*t* test (*df*)		*P* value^a^		Cohen *d*	
TUG^b^					–1.63 (–3.19 to –0.07)						0.59
	Experimental group	13.11 (4.39 )	9.95 (2.41)			3.46 (24)		.001			
	Control group	12.78 (3.35)	11.58 (3.04)			2.42 (24)		.02			
	*t* test (*df*)	–0.33 (48)	–2.11			—^c^		—			
	*P* value	.74	.04			—		—			
Stand on 1 foot					–5.96 (–33.2 to 21.3)						0.13
	Experimental group	24.79 (19.71)	43.72 (31.59)			4.14 (24)		<.001			
	Control group	38.36 (79.30)	49.68 (58.19 )			2.52 (24)		.01			
	*t* test (*df*)	–0.18 (48)	–0.44 (48)			—		—			
	*P* value	.86	.66			—		—			
30-second CST^d^					1.28 (–1.18 to 3.74)						0.31
	Experimental group	9.52 (3.55)	13.96 (4.94)			–5.73 (24)		<.001			
	Control group	11.16 (3.88)	12.68 (3.00)			–2.63 (24)		.02			
	*t* test (*df*)	–1.39 (48)	–0.78 (48)			—		—			
	*P* value	.16	.43			—		—			
FTSST^e^					–0.16 (–1.67 to 1.35)						0.06
	Experimental group	13.56 (6.78)	9.32 (2.90)			3.79 (24)		<.001			
	Control group	11.03 (3.00)	9.48 (2.24 )			2.35 (24)		.02			
	*t* test (*df*)	–1.45 (48)	0.25 (48)			—		—			
	*P* value	.15	.80			—		—			
Two-minute step test					2.36 (–17.8 to 22.5)						0.07
	Experimental group	132.56 (41.46)	179.84 (38.27)			–6.10 (24)		<.001			
	Control group	143.88 (33.74)	177.48 (30.25)			–4.82 (24)		<.001			
	*t* test (*df*)	–0.98 (48)	0.24 (48)			—		—			
	*P* value	.33	.81			—		—			

^a^The significance level was set at *P*<.05.

^b^TUG: Timed Up and Go.

^c^Not available.

^d^30-second CST: 30-second Chair-Stand Test.

^e^FTSST: Five-times Sit-To-Stand Test.

### Comparison of Quality of Life Indicators Between the 2 Groups of Patients

At baseline, there were no statistically significant differences between the experimental and control groups across all FACT-L subscales and total score (all *P*>.05), indicating good comparability before intervention. For PWB, the experimental group improved from 14.68 (SD 2.27) to 21.68 (SD 2.27; *T*=–4.55; *P*<.001), while the control group improved from 13.72 (SD 2.23) to 17.68 (SD 2.27; *T*=–4.52; *P*<.001). The between-group comparison revealed a statistically significant difference favoring the motion capture cohort (mean difference 4.00 points, 95% CI 2.66-5.34; *T*=6.24; *P*<.001). For SWB, the experimental group improved from 16.04 (SD 1.24) to 20.08 (SD 1.32; *T*=–4.33; *P*<.001), and the control group improved from 16.80 (SD 1.53) to 19.80 (SD 1.38; *T*=–4.40; *P*<.001). However, the between-group difference was not statistically significant (mean difference 0.28 points, 95% CI –0.76 to 1.32; *T*=–0.53; *P*=.60). For EWB, the experimental group improved from 13.48 (SD 1.16) to 15.80 (SD 1.19; *T*=–4.48; *P*<.001), and the control group improved from 13.72 (SD 1.49) to 15.92 (SD 1.38; *T*=–4.44; *P*<.001). The between-group difference did not reach statistical significance (mean difference –0.12 points, 95% CI –0.98 to 0.74; *T*=–0.27; *P*=.79). For FWB, the experimental group improved from 12.28 (SD 2.23) to 18.68 (SD 2.27; *T*=–4.55; *P*<.001), while the control group improved from 11.04 (SD 2.25) to 14.00 (SD 1.94; *T*=–4.44; *P*<.001). The between-group comparison revealed a statistically significant difference favoring the motion capture cohort (mean difference 4.68 points, 95% CI 3.44-5.92; *T*=7.85; *P<.*001)*.* For the Additional Concerns–Lung (ACL) subscale, the experimental group improved from 9.72 (SD 1.93) to 17.24 (SD 2.11; *T*=–4.41; *P*<.001), and the control group improved from 9.64 (SD 1.91) to 12.96 (SD 1.77; *T*=–4.41; *P*<.001). The between-group difference was statistically significant (mean difference 4.28 points, 95% CI 3.14-5.42; *T*=7.78; *P<.*001). For the total FACT-L score, the experimental group improved from 66.20 (SD 6.83) to 93.36 (SD 6.72; *T*=–51.12; *P*<.001), while the control group improved from 64.92 (SD 6.85) to 80.36 (SD 6.04; *T*=–33.94; *P*<.001). The between-group comparison revealed a statistically significant difference favoring the experimental group (mean difference 13.00 points, 95% CI 9.30-16.70; *T*=7.19; *P*<.001). Complete data are provided in Table 7.

**Table 7:** Comparison of quality of life indicators before and after intervention between the 2 groups.

Index and group		Pre-exercise, mean (SD)	Postexercise, mean (SD)	Between-group difference (95% CI)		*t* test (*df*)		*P* value^a^		Cohen *d*	
PWB^b^					4.00 (2.66-5.34)						1.76
	Experimental group	14.68 (2.27)	21.68 (2.27)			–4.55 (24)		<.001			
	Control group	13.72 (2.23)	17.68 (2.27)			–4.52 (24)		<.001			
	*t* test (*df*)	1.51 (48)	6.24			—^c^		—			
	*P* value	.14	<.001			—		—			
SWB^d^					0.28 (–0.76 to 1.32)						0.21
	Experimental group	16.04 (1.24)	20.08 (1.32 )			–4.33 (24)		<.001			
	Control group	16.80 (1.53)	19.80 (1.38)			–4.40 (24)		<.001			
	*t* test (*df*)	–1.79 (48)	–0.53			—		—			
	*P* value	.07	.60			—		—			
EWB^e^					–0.12 (–0.98 to 0.74)						0.09
	Experimental group	13.48 (1.16)	15.80 (1.19)			–4.48 (24)		<.001			
	Control group	13.72 (1.49)	15.92 (1.38)			–4.44 (24)		<.001			
	*t* test (*df*)	–1.77 (48)	–0.27			—		—			
	*P* value	.08	.79			—		—			
FWB^f^					4.68 (3.44 to 5.92)						2.22
	Experimental group	12.28 (2.23)	18.68 (2.27)			–4.55 (24)		<.001			
	Control group	11.04 (2.25)	14.00 (1.94)			–4.44 (24)		<.001			
	*t* test (*df*)	1.96 (48)	7.85			—		—			
	*P* value	.06	<.001			—		—			
ACL^g^					4.28 (3.14 to 5.42)						2.20
	Experimental group	9.72 (1.93)	17.24 (2.11 )			–4.41 (24)		<.001			
	Control group	9.64 (1.91)	12.96 (1.77 )			–4.41 (24)		<.001			
	*t* test (*df*)	–0.72 (48)	7.78			—		—			
	*P* value	.47	<.001			—		—			
Total score					13.00 (9.30 to 16.70)						2.03
	Experimental group	66.20 (6.83 )	93.36 (6.72 )			–51.12 (24)		<.001			
	Control group	64.92 (6.85)	80.36 (6.04)			–33.94 (24)		<.001			
	*t* test (*df*)	0.66 (48)	7.19			—		—			
	*P* value	.51	<.001			—		—			

^a^The significance level was set at *P*<.05.

^b^PWB: physical well-being.

^c^Not available.

^d^SWB: social/family well-being.

^e^EWB: emotional well-being.

^f^FWB: functional well-being.

^g^ACL: Additional Concerns-Lung subscale.

### Comparison of Treatment Compliance and Incidence of Adverse Events Between the 2 Groups of Patients

Home-based program adherence was markedly greater in the motion capture group (18/25, 72% vs 10/25, 40%; *P*=.02), and no exercise-related adverse events were recorded in either group (Table 8).

**Table 8:** Comparison of treatment compliance between the 2 groups.

Group	Training compliance rate (≥3 times per week)	Noncompliance rate (≤3 times per week)
Experimental group, n (%)	18 (72)	7 (28)
Control group, n (%)	10 (40)	
Test value	5.180	—^a^
*P* value	.02	—

^a^Not available.

## Discussion

### Principal Findings

This study demonstrated that motion recognition–based telerehabilitation was associated with greater improvements in postoperative recovery among patients with NSCLC compared with conventional home-based video-guided training. Specifically, participants in the intervention group showed superior gains in exercise tolerance, pulmonary function, selected aspects of functional mobility, and the physical and functional domains of quality of life. Rehabilitation adherence was also higher in the motion-guided group, and no intervention-related adverse events were observed in either group.

Taken together, these findings suggest that integrating real-time kinematic feedback into home-based pulmonary rehabilitation may enhance movement quality, functional recovery, and patient engagement beyond standard remote instruction. Importantly, these results are consistent with emerging evidence from recent systematic reviews and network meta-analyses [27] indicating that telerehabilitation can achieve improvements in physical function and quality of life comparable with, or in some contexts exceeding, conventional face-to-face pulmonary rehabilitation programs. This study extends this body of evidence by highlighting the potential added value of motion recognition–based feedback within a postoperative population with lung cancer.

### Impact of Telerehabilitation Based on Motion Recognition Technology on Exercise Tolerance in Postoperative Patients With NSCLC

Exercise tolerance constitutes a primary indicator of postoperative functional recovery in patients undergoing NSCLC resection [28]. Baseline 6MWD did not differ between groups, confirming equivalent preintervention capacity. Although both cohorts achieved after-intervention gains—reflecting the efficacy of any structured training—the experimental group demonstrated a substantially larger increment, underscoring the supplementary value of motion capture–guided telerehabilitation. These observations align with prior evidence.

The superior improvement in the motion capture group can be attributed to several interconnected mechanisms. First, real-time kinematic feedback facilitates motor learning through augmented feedback loops. According to the guidance hypothesis (“Comparative Effects of Sensory Motor and Virtual Reality Interventions to Improve Gait, Balance, and Quality of Life MS Patients”), concurrent feedback enhances skill acquisition by providing immediate error information, allowing patients to correct aberrant movement patterns (eg, knee valgus and excessive trunk sway) before they become ingrained. This process engages the cerebellar-cortical circuit, promoting neuroplastic changes that optimize movement efficiency [29].

Second, real-time, AI-guided motion correction may augment walking economy by refining neuromuscular synchrony and dynamic stability. Earlier work documents that cancer survivors exhibit quadriceps weakness approximately one-third to two-fifths greater than healthy peers [30]. Instantaneous joint-angle feedback during tasks such as squats promptly redirects aberrant mechanics—knee valgus or excessive trunk sway—reducing metabolic cost per stride.

Third, patients in the experimental group covered more ground during the 6MWT without parallel rises in Borg ratings; superior gait efficiency and balance translated into greater distance with lower perceived exertion. Moreover, quantitative visual cues (eg, “posture accuracy: 92%”) reinforced engagement and intensity, yielding a compliance rate of 72% (18/25) versus 40% (10/25) in controls. Sustained participation drove cumulative cardiopulmonary and musculoskeletal adaptations—the principal determinants of improved exercise capacity [31].

The Borg scale gauges perceived exertion and complements objective measures of exercise tolerance [32]. In our trial, Borg ratings did not differ between the motion capture and control groups. Nevertheless, instantaneous kinematic feedback appeared to bolster self-efficacy and refine movement efficiency, allowing the experimental cohort to increase walking distance without proportionate fatigue. Conversely, controls—hampered by suboptimal mechanics—covered shorter distances and yet reported greater perceived exertion per meter.

### Impact of Telerehabilitation Based on Motion Recognition Technology on Pulmonary Function in Postoperative Patients With NSCLC

Enhancing pulmonary function represents a key objective in postoperative care for patients with lung cancer [33]. The superior improvements in FVC, FEV_1_, and PEF observed in the motion capture group likely stem from the system’s capacity to ensure optimal respiratory muscle synergy through real-time corrective feedback. While participants in both cohorts completed identical training protocols, only those in the intervention group received real-time monitoring of biomechanical parameters with prompt adjustments for aberrant movement patterns [34].

While participants in both cohorts completed identical exercise protocols, only those in the intervention group received continuous monitoring of trunk alignment during core exercises. This distinction is critical because respiratory function is intimately linked to core stability. The diaphragm, the primary muscle of inspiration, shares fascial connections with the transversus abdominis and pelvic floor muscles, forming the thoracoabdominal pump [35]. Proper activation of this integrated system requires precise coordination—a skill that benefits from augmented feedback.

Consider the plank—an evidence-based core stabilization maneuver engaging key musculature including the transversus abdominis, rectus abdominis, and erector spinae. Regular plank training (3 times per week) enhances both respiratory function and general fitness in aging populations [36]. Yet, its benefits are substantially attenuated by technical errors such as hyperlordosis [37], which specifically impairs transversus abdominis recruitment [38]. Such deviations not only undermine therapeutic effects but also elevate spinal loading and injury potential.

The motion recognition system provided instantaneous proprioceptive feedback that helped patients maintain neutral spine alignment, thereby optimizing the feedforward control mechanism of core muscles. This feedforward activation—where trunk muscles contract in anticipation of limb movement—is essential for maintaining spinal stability during dynamic activities [39]. By reinforcing this mechanism during training, the intervention likely enhanced the neural drive to respiratory muscles, translating into improved spirometric outcomes. Furthermore, the observed improvements in PEF suggest enhanced expiratory muscle function, possibly through better recruitment of abdominal muscles during forced expiration.

### Impact of Telerehabilitation Based on Motion Recognition Technology on Functional Mobility in Postoperative Patients With NSCLC

Functional mobility assessment serves as a critical component in evaluating rehabilitation success. Standardized tests such as the TUG [40], single-leg balance assessment [41], 30-second CST [42], FTSST [43], and 2-minute stepping task [44] collectively offer a comprehensive evaluation of movement capacity and functional performance. These validated measures enable clinicians to objectively determine patients’ functional capacity for activities of daily living and community participation, while effectively tracking recovery milestones.

Among these assessments, the TUG represents a composite functional task requiring coordinated execution of multiple motor components, including sit-to-stand transition, gait initiation, turning, and resitting. Performance on this test reflects not only lower limb strength but also dynamic balance, motor sequencing, and intertask coordination [45]. The observed improvement in TUG performance in the motion recognition group may be related to enhanced movement precision and temporal coordination facilitated by real-time kinematic feedback. The motion recognition system provides continuous monitoring of joint angles and movement trajectories, potentially supporting more accurate sensorimotor integration during exercise execution—the process by which sensory feedback refines motor output [46]. Similar improvements in postural control and motor stability have been reported in neurological populations receiving telemedicine-based rehabilitation, including individuals with Parkinson disease, suggesting that remote feedback mechanisms may enhance movement regulation across different clinical conditions [47]. Real-time visual feedback of joint angles and movement trajectories may have facilitated implicit motor learning, whereby patients internalize correct movement patterns without extensive conscious processing. This contrasts with explicit learning approaches that rely primarily on verbal instruction and are more cognitively demanding and potentially less durable under stress [48]. The improved TUG performance in the experimental group likely reflects enhanced procedural memory for the sequence of movements involved in the test.

The lack of significant between-group differences in single-leg stance, 30-second CST, FTSST, and 2-minute step tests does not contradict this interpretation. These tests predominantly assess isolated components of physical function—static balance, lower extremity strength, and repetitive endurance—rather than the integrated motor control required for complex tasks. Both groups received identical exercise prescriptions targeting lower limb musculature, likely producing comparable gains in gross strength (as reflected in the similar improvements on these tests). The unique value of the motion-aware system lies in optimizing movement quality and interjoint coordination—domains captured by composite measures such as the TUG but not by simpler functional tests. This dissociation between strength gains and coordination improvements is consistent with the principle that motor skill acquisition and muscular adaptation are mediated by distinct neural and physiological mechanisms [49].

### Impact of Telerehabilitation Based on Motion Recognition Technology on Quality of Life in Postoperative Patients With NSCLC

Quality of life represents a critical multidimensional end point in postsurgical NSCLC rehabilitation, encompassing physical, psychological, and social well-being as primary markers of recovery success [50]. Our intervention group showed statistically superior improvements in PWB, FWB, ACL, and the total FACT-L score relative to controls. This pattern implies that motion-aware telerehabilitation offers particular advantages for restoring physical and functional aspects of quality of life, potentially attributable to its capacity for optimizing functional recovery through millimeter-precise movement analysis and instantaneous form correction. Additionally, the system’s ability to provide symptom-specific movement modifications and breathing technique guidance may explain this therapeutic advantage, empowering patients to better control their condition-related challenges.

The analysis revealed no statistically significant intergroup differences in either SWB or EWB domain. This observation may be explained by several key considerations. First, meaningful enhancement of social support systems and psychological state typically necessitates prolonged intervention durations and often benefits from dedicated mental health support, posing challenges for demonstrating immediate effects within a limited study period [51]. Second, the psychological status of postsurgical patients with NSCLC is frequently influenced by complex psychosocial factors—particularly disease recurrence anxiety and treatment-related financial stress [52]—that extend beyond the scope of physical rehabilitation and may require targeted psychotherapeutic approaches combined with robust social services.

### Impact of Telerehabilitation Based on Motion Recognition Technology on Exercise Adherence and Safety in Postoperative Patients With NSCLC

Program adherence represents a critical determinant of rehabilitation efficacy [53]. Our data revealed substantially better compliance rates in the intervention group than in controls, indicating that motion-aware telerehabilitation systems may optimize treatment engagement for postsurgical patients with NSCLC. This effect appears mediated by the platform’s multimodal feedback mechanisms—including real-time performance metrics, achievement scoring, and graphical progress tracking—which collectively enhance treatment motivation through positive reinforcement. Contemporary research confirms that instantaneous performance feedback bolsters rehabilitation adherence by reinforcing patient self-confidence and perceived competence [54]. Furthermore, the system’s interactive design elements convert conventional exercises into dynamic, goal-oriented tasks, offering superior psychological engagement compared with passive video demonstrations.

Regarding safety outcomes, neither study group reported intervention-related adverse events, confirming the acceptable risk profile of home-based motion capture rehabilitation. The technology’s ability to continuously monitor and correct biomechanical execution helps ensure proper movement patterns, consequently minimizing injury risks associated with faulty technique [55].

### Limitations

Despite the promising findings, several constraints warrant consideration. First, assessments were restricted to the 4-week end point, precluding insight into durability; subsequent studies should extend follow-up to 3, 6, and 12 months to chart longer-term recovery. Second, the modest sample size may limit both external validity and statistical power. Although significant between-group differences were observed for several primary outcomes, the study may have been underpowered to detect smaller yet clinically meaningful effects in some secondary measures. Accordingly, the absence of statistically significant differences for certain functional indicators should be interpreted cautiously, as the possibility of type II error cannot be excluded. Larger, adequately powered multicenter trials are required to validate and refine these findings. Third, the lack of preoperative functional baselines hampers delineation of the full perioperative trajectory; future protocols should incorporate presurgical assessments to construct comprehensive longitudinal recovery profiles.

### Conclusions

To our knowledge, this is the first randomized controlled trial to integrate motion capture analytics into telerehabilitation following NSCLC surgery. Over 4 weeks, the approach proved both safe and superior to standard video-guided exercise, yielding larger gains in exercise tolerance, spirometry, functional mobility, and quality of life. These data establish a clinically viable strategy that can readily be scaled across telemedicine and postoperative care pathways.

## Data Availability

The datasets used and/or analyzed during this study are available from the corresponding author on reasonable request.

## Appendix

Multimedia Appendix 1 CONSORT-EHEALTH (Consolidated Standards of Reporting Trials of Electronic and Mobile Health Applications and Online TeleHealth) (V 1.6.1) checklist.

Multimedia Appendix 2 The pulmonary function test graph and the 6-minute walk test graph.

## Abbreviations

ACL: Additional Concerns–Lung
AI: artificial intelligence
CST: Chair-Stand Test
EWB: emotional well-being
FACT-L: Functional Assessment of Cancer Therapy–Lung v4.0
FEV1: forced expiratory volume in 1 second
FTSST: Functional Mobility: Five-times Sit-to-Stand Test
FVC: forced vital capacity
FWB: functional well-being
NSCLC: non–small cell lung cancer
PEF: peak expiratory flow
PWB: physical well-being
6MWD: 6-minute walk distance
6MWT: 6-minute walk test
SWB: social/family well-being
TUG: Timed Up and Go
