# Supplementary figures and images for: Effects of Telerehabilitation Based on Motion Recognition Technology on Exercise Endurance of Patients With Non–Small Cell Lung Cancer After Surgery: Single-Center, Prospective, Open-Label, Randomized Controlled Trial
Source: JMIR Mhealth Uhealth. 2026 Jun 29;14:e82447. doi: 10.2196/82447 (PMC13365894; doi:10.2196/82447)

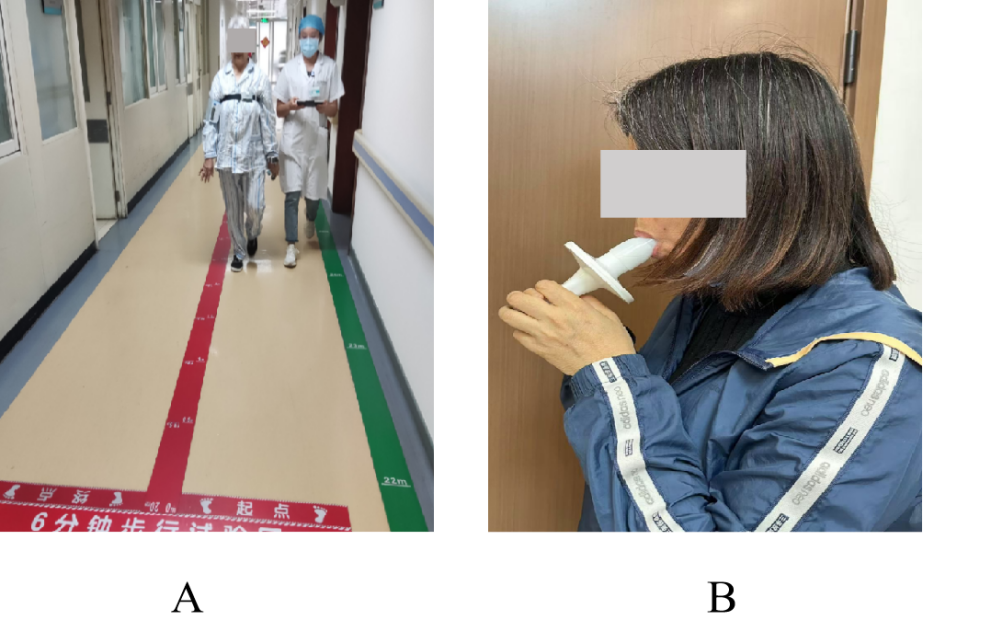


(A) Six-minute walking test; (B)pulmonary function test diagram

Supplement: Multimedia Appendix 2 [file mhealth_v14i1e82447_app2.doc]
